# The impact of a short-term training program on workers’ sterile processing knowledge and practices in 12 Ethiopian hospitals: A mixed methods study

**DOI:** 10.1371/journal.pone.0215643

**Published:** 2019-05-01

**Authors:** Olive M. Fast, Hareya Gebremedhin Teka, Mussie Alemayehu/Gebreselassie, Christina Marie Danielle Fast, Dan Fast, Faith-Michael E. Uzoka

**Affiliations:** 1 Sterile Processing Education Charitable Trust (SPECT), Calgary, Canada; 2 College of Health Science, Mekelle University, Mekelle, Ethiopia; 3 Department and Mathematics and Computing, Mount Royal University, Calgary, Canada; Cincinnati Children's Hospital Medical Center, UNITED STATES

## Abstract

**Background:**

The need for increased attention to surgical safety in low- and middle-income countries invited organizations worldwide to support improvements in surgical care. However, little is written about issues in instrument sterilization in low- and middle-income countries including Ethiopia.

**Objective:**

The study aims to identify the impact of a sterile processing course, with a training-of-trainers component and workplace mentoring on surgical instrument cleaning and sterilization practices at 12 hospitals in Ethiopia.

**Method:**

A mixed-methods research design that incorporates both qualitative and quantitative research approaches to address issues in sterile processing was used for this study. The quantitative data (test results) were validated by qualitative data (hospital assessments, including observations and participant feedback). Twelve hospitals were involved in the training, including two university teaching hospitals from two regions of Ethiopia. In each of the two regions 30 sterile processing staff were invited to participate in a three-day course including theory and skills training; 12–15 of these individuals were invited to remain for a two-day training of trainers course. The collected quantitative data were analysed using a paired t-test by SPSS software, whereas comparative analysis was employed for the qualitative data.

**Results:**

Process, structural, and knowledge changes were identified following program implementation. Knowledge test results indicated an increase of greater than 20% in participant sterile processing knowledge. Changes in process included improved flow of instruments from dirty to clean, greater attention to detail during the cleaning and decontamination steps, more focused inspection of instruments and careful packaging, as well as changes to how instruments were stored. Those trained to be trainers had taught over 250 additional staff.

**Conclusions:**

Increased attention to and knowledge in sterile processing practices and care of instruments with a short, one-week course provides evidence that a small amount of resources applied to a largely under-resourced area of healthcare can result in decreased risks to patients and staff. Providing education in sterile processing and ensuring staff have the ability to disseminate their learnings to other health care providers results in decreasing risks of hospital associated infections in patients.

## Introduction

A 2014 publication in the Lancet [1] highlighting the need for increased attention to surgical safety in low- and middle-income countries (LMIC’s) invited organizations worldwide to support improvements in surgical care. Subsequent to this publication the World Health Assembly passed a resolution in 2015 to strengthen essential surgical care as a component of universal health coverage [2]. The resolution included a call for strengthening of infection prevention and control standards to ensure safety in surgical care. Infection control standards include ensuring the sterility of surgical instruments. The World Health Organization (WHO) acknowledges that ensuring sterility and safety of surgical instruments involves having adequate resources, education/training in sterile processing (SP), and appropriate policies/procedures governing SP practices [3].

There is little written about issues in instrument sterilization in LMICs, however barriers have been identified that include lack of resources, education/training and policies/procedures [3–7]. O’Hara et al -[5]- found at least one sterility parameter variable was not within target range in all (n = 9) responding LMIC sites involved in their study of sterilization practices. In their qualitative analysis of interviews with staff at two LMIC facilities, Aveling et al -[6]- identified a lack of resource availability and functionality, as well as poor adherence to or availability of policies/procedures. In an analysis of 59 facilities in 3 LMICs Fast et al-[7]- highlighted the absence of cleaning supplies, including basic brushes and enzymatic detergents, as well as deficiencies in sterilizing equipment (such as functioning autoclaves, distilled water, adequate electricity). Of significance in Fast et al finding was that not one of the 59 facilities surveyed “were able to adhere to even basic WHO standards for sterile processing” (p.6), indicating that all estimated annual 80,000 surgeries occurred under unsterile conditions. While WHO (2016) standards recommend staff have formal education in SP, only 12% of SP staff in 2 of 3 countries (the 3^rd^ country had none) were found to have formal training [7].

In efforts to address the SP education gap in LMICs, Sterile Processing Education Charitable Trust (SPECT) implemented a training course in Benin. A mixed-methods study evaluating the impact of this SP course on decreasing the risks of surgical care identified a significant impact on workers’ knowledge, understanding of their work, attitudes towards their work, and perceived impact on practice [8]. Participants noted an increased ability to identify appropriate resources to support their work as well as an increased understanding of how their work impacted patients. However, we have limited evidence related to sterile processing in LMICs, including Ethiopia. In light of this, the study aimed to identify the impact an education program focused on instrument sterilization and medical device reprocessing had on 12 health care facilities in Ethiopia.

## Methods

This study sought to identify the impact of a SP training program, including theory lectures, a training of the trainers (ToT) component and workplace mentoring for health care workers (HCWs) from12 Safe Surgery 2020 [9] hospitals in Ethiopia. The study was approved by Mekelle University Human Research Ethics Review Committee (ERC 1047/2017) and conforms to the principles embodied in the Declaration of Helsinki. The study was explained to participants in English and Amharic and informed written consent obtained in English.

A mixed-methods research design [10] that incorporates both quantitative and qualitative research approaches to address the impact of reprocessing surgical instruments was used for this study. The quantitative data (test results) were validated by qualitative data (hospital assessments, including observations and participant feedback) to strengthen study findings [10–11].

From February to October 2017 the research team collaborated with the Ethiopian Ministry of Health, SPECT, and Assist International to implement a SP training program for HCWs from 12 hospitals in 2 regions of Ethiopia involved in a Safe Surgery 2020 (SS2020) initiative. SS2020 is a collaboration of foundations, nonprofits, educational institutions and local governments who want to make surgery safe, affordable and accessible throughout the world, and is funded by the GE Foundation [9]. The two University teaching hospitals and 10 smaller hospitals involved in the study had a total of 39 operating rooms, where 238–269 surgeries were performed weekly (Table 1).

**Table 1:** Demographic data.

Pre-assessment date	Feb/March 2017
Post-assessment date	Nov 2017
Number of operating rooms	35–39
Surgeries performed weekly	238–269
Number of staff members who received more than one hour of classroom training on SP **pre**-SPECT training	4
Number of staff members who received more than one hour of classroom training on SP **post**-SPECT training	38
Number of staff members who received on-the-job training on SP **pre**-SPECT training	83
Number of staff members who received on-the-job training on SP **post**-SPECT training	283

### Intervention description

After the initial consultation with the Ministry of Health to identify Safe Surgery 2020 hospitals, baseline hospital assessments (S1 Appendix) of SP practices were conducted using a Hospital SP Assessment tool [8]. Data collected included pre- and post-training hospital assessments as well as pre- and post-training participant tests S2 Appendix). Hospital administrators were invited to send SP workers to the training. Inclusion criteria for the course consisted of those involved in the SP practices or those who were responsible for overseeing the SP process.

Two short term courses were provided, one in April 2017 in the Tigray Region and another in July 2017 in the Amhara Region. Medical education was provided in English in Ethiopia; therefore, all training was done in English with support by an Amharic speaking translator as needed. Participants received three days of classroom training. Lecture topics included microbiology, infection prevention and control, cleaning and decontamination, disinfection, instrument inspection and packaging, sterilization, sterile storage, and transportation. All participants wrote a SP knowledge test before training and again at the end of the third day. Following the fundamentals course select participants from each hospital were chosen, based on their improvement in post-training test scores and course engagement, to participate in a two-day ToT course to strengthen their ability to teach the subject matter to others.

Following the training program each participating hospital received on-site visits by the SPECT educator and recommendations were made regarding how SP processes could be improved. Following the educator’s mentoring visits, hospital post assessments were done in the Tigray (four-months post educator visit) and Amhara (six-months post visit) regions.

### Data analysis

Data from hospitals were identified by region (alphabetically) and number (1–6), and participants identified numerically. Data were analyzed using a concurrent embedded mixed methods approach [10]. Quantitative data from participant knowledge tests and hospital assessments were analyzed (in SPSS statistical software) using paired t-test and chi square (χ^2^) analysis to test the null hypothesis that there were no statistical differences in the variables under investigation after the training at 0.05 level of significance. The chi-square test was applied to individual variables under consideration, while the t-test was used to test the aggregate effect of the training on workers’ SP knowledge and practices. In both tests, the improvements were considered significant if the p ≤ 0.05. Clinical significance of our results was also determined using minimal clinically important differences (MCID) and relative clinical effectiveness (RCE). Hospital SP Assessment Tool qualitative data were analyzed using comparative analysis [12].

## Results

We report on comparisons between the two separate data sets–pre- and post-hospital assessments and pre- and post-test results—to identify changes in SP knowledge and practices [11].

### Knowledge test result

53 participants from the 12 hospitals completed both the pre-course and post-course SP knowledge tests. Analysis of test scores show significant improvements in participants’ SP knowledge acquisition. The mean pre-course test score was 19.30 (±4.65) and mean post-course 27.77 (±6.99). The mean difference was 8.47 at t-value 10.574 (p<0.001) (Table 2).

**Table 2:** SP training participants pre-post test results in ethiopian hospitals, 2017.

Paired sample	Sample size	Mean-pre-test	Std Dev	Mean-post-test	Std Dev	Correlation	t-value	df	Sig (2-tail)	Range-post-test
	53	19.30	4.65	27.77	6.99	.562	10.574	52	.001	14–39

### Hospital SP assessments

As shown in Table 3, statistically significant improvement was found in several areas of SP processes post training. These areas included: improved scheduling of regular SP room cleaning (P-value 0.027), increased availability of posters on the walls providing instruction to staff working in SP rooms (p-value 0.012), increased use of clean brushes in good condition (p-value 0.034), increased soaking of instruments in soap and water as opposed to 0.5% chlorine solution immediately after surgery (p-value 0.001), increased access to clean, lint free cloths (p-value 0.011), and increased careful inspection of instruments prior to packaging (p-value 0.002). A marginally significant improvement in the use of personal protective equipment by SP staff (p-value 0.059) was also identified.

**Table 3:** SP hospital pre-post assessment results in ethiopian hospitals, 2017.

Variable	Pre-Assessment	Post-Assessment	X2	P-value
SP area cleaned on scheduled rotating basis	5(41.6%)	11(91.6%)	6.5	0.027
Posters on the wall providing instruction to SP staff	2(16.6%)	9(75%)	7.8	0.012
Clean brushes in good condition in use in the decontamination area	2(16.7%)	5(41.7%)	4.5	0.034
Instruments soaked in soap and water immediately after use in the operating room	0(0%)	10(83.3%)	16.4	0.001
SP staff have access to clean, lint free cloths, various sizes	0(0%)	5(41.6%)	6.31	0.011
Instruments carefully inspected prior to packaging	1(8.3%)	9(75%)	10.51	0.002

While the above noted indicators showed statistically significant improvement post-training, other indicators showed no statistically significant improvement. Of those showing no statistically significant improvement, initial high scores were identified in five indicators: use of a sharps container, incineration of sharps on the premises, rinsing of instruments in clean water after removal of soil, and use of more than one sink in the cleaning process. There had already been good adherence to these variables before training and thus little change was noticed after training.

Three indicators showing no improvement involved supplies that were virtually unavailable to participating hospitals but are regularly used in high-income countries. These included use of enzymatic detergents, disinfectants and biological indicators. Table 4 identifies areas of assessment where a small increase in the number of hospitals engaging in SP practices where noted, but were statistically insignificant.

**Table 4:** Statistically insignificant improvements to sp practices pre-post assessment in ethiopian hospitals, 2017.

Variable	Pre-Assessment	Post-Assessment	X2	P-value
There are signs restricting access of unauthorized staff to the SP area	4(33.3%)	6(50%)	1.000	0.317
Soap available for hand washing for SP staff	8(66.7%)	10(83.3%)	0.500	0.480
Running water available for hand washing for SP staff	7(58.3%)	11(91.7%)	2.286	0.131
Contaminated instruments flow from dirty to clean areas in the SP process	4(33.3%)	7(58.3%)	2.250	0.134
Instruments moved to a clean area with no dirty instruments after cleaning and drying	8(66.7%)	7(58.3%)	0.125	0.724
Functional autoclave being used to sterilize instruments	8(66.6%)	10(83.3%)	0.500	0.480
Autoclave/dry heat sterilizer in a room separate from the decontamination area	7(58.3%)	8(66.7%)	0.143	0.706
Chemical indicators being used	5(41.7%)	8(66.7%)	1.800	0.277
Times posted indicating when instruments were placed in sterilizers	1(8.3%)	2(16.7%)	1.000	0.317
Instruments stored in an enclosed area after sterilization	9(75%)	8(66.7%)	0.111	0.739
Sterilized instruments stored away from decontamination areas	7(58.3%)	8(66.7%)	0.143	0.705
SP staff wear personal protective equipment	8(66.7%)	11(91.7%)	1.125	0.289

Statistical significance enables us to test the statistical hypothesis that there is a significant difference in sterile processing practices pre- and post-training; however, this does not imply clinical significance. A study outcome can be statistically significant, but not clinically significant and vice versa [13]. Our current study is inherently underpowered due to the sample size of hospitals (<30) according to the law of large numbers [14,15]. In this case you might fail to detect an important difference between groups. We recorded statistically significant improvements on six variables (Table 3) and statistically insignificant effects on twelve variables (Table 4). Determining clinical significance is highly subjective; however, we relied on the guideline provided in [16] to generate a table of clinical significance (Table 5). To determine clinically relevant changes in outcomes, the ‘minimal clinically important differences (MCID)’ were computed as follows:

**Table 5:** Clinical significance test.

Variable	Pre-Assessment	Post-Assessment	MCID	Clinical effect
SP area cleaned on scheduled rotating basis	5(41.6%)	11(91.6%)	1.863701	large (+)
Posters on the wall providing instruction to SP staff	2(16.6%)	9(75%)	2.174318	large(+)
Clean brushes in good condition in use in the decontamination area	2(16.7%)	5(41.7%)	0.931851	large(+)
Instruments soaked in soap and water immediately after use in the operating room	0(0%)	10(83.3%)	3.106169	large(+)
SP staff have access to clean, lint free cloths, various sizes	0(0%)	5(41.6%)	1.553084	large(+)
Instruments carefully inspected prior to packaging	1(8.3%)	9(75%)	2.484935	large(+)
There are signs restricting access of unauthorized staff to the SP area	4(33.3%)	6(50%)	0.621234	moderate(+)
Soap available for hand washing for SP staff	8(66.7%)	10(83.3%)	0.621234	moderate(+)
Running water available for hand washing for SP staff	7(58.3%)	11(91.7%)	1.242468	large(+)
Contaminated instruments flow from dirty to clean areas in the SP process	4(33.3%)	7(58.3%)	0.931851	large(+)
Instruments moved to a clean area with no dirty instruments after cleaning and drying	8(66.7%)	7(58.3%)	-0.31062	small(-)
Functional autoclave being used to sterilize instruments	8(66.6%)	10(83.3%)	0.621234	moderate(+)
Autoclave/dry heat sterilizer in a room separate from the decontamination area	7(58.3%)	8(66.7%)	0.310617	small(+)
Chemical indicators being used	5(41.7%)	8(66.7%)	0.931851	large(+)
Times posted indicating when instruments were placed in sterilizers	1(8.3%)	2(16.7%)	0.310617	small(+)
Instruments stored in an enclosed area after sterilization	9(75%)	8(66.7%)	-0.31062	small(-)
Sterilized instruments stored away from decontamination areas	7(58.3%)	8(66.7%)	0.310617	small(+)
SP staff wear personal protective equipment	8(66.7%)	11(91.7%)	0.931851	large(+)

MCID = Change in treatment values/ pooled standard deviation of pre-and post-treatment

MCID is a directional value, which is evaluated as follows: <0.2 = trivial effect; 0.2–0.5 = small effect; 0.5–0.8 = moderate effect; > 0.8 = large effect.

The MCID provides better clinical information to make a valuable judgment on the clinical significance of the effects of the training on SP practices in the hospitals. As expected, all six variables that showed statistical significance also exhibited large positive clinical significance. In addition, three other variables that did not show statistical significance exhibited clinical significance, while three others showed moderate clinical significance. The overall clinical effectiveness of the treatment (training) could be determined by computing the relative clinical effectiveness (RCE) value [13] based on an expected moderate or above effect using the following formula:

$$Relative\;clinical\;effectiveness=\frac{\%\;with\;clinically\;meaningful\;outcome\;}{\%\;without\;clinically\;meaningful\;outcome\;}$$

Based on the data in Table 5, the RCE = (13/18)/(5/18) = 2.6. A relative clinical effectiveness of 1.0 is considered 50% successful. Our study shows a very high RCE of 2.6, which is extremely successful.

#### Use of supplies and equipment

While all 12 (100%) hospitals used 0.5% chlorine solution in the instrument decontamination process pre-training, as required by the Ethiopian infection control manual, 8 (68%) hospitals had removed chlorine from the process post training. The remaining 4 (32%) had decreased the time instruments were soaked in the chlorine solution to 10 minutes or less post training. Pre-training 12 (100%) hospitals used large brushes in poor condition to clean instruments, whereas post-training 4 (33%) hospitals were noted to have brushes in good condition, while one hospital’s brushes had deteriorated since training and not been replaced. At one hospital brushes donated by SPECT were stored away while staff were required to continue using brushes in poor condition that were too large to be effective and in poor condition.

#### Functional changes

Post-training 3 (25%) hospitals had made functional changes. One added a new sink, another put a table in the decontamination room for basins in which instruments could be cleaned (replacing buckets on the floor), and a third implemented the three-sink method for cleaning. A different facility was found to continue storing sterilized instruments in the decontamination area post-training, while another had not yet implemented a recommendation to separate the operating theatre from the sterilization area with the use of a plastic curtain.

#### Flow of instruments

Recommendations for SP are that instruments have a uni-directional flow from dirty to clean areas without cross-contamination. Pre-training 4 (33%) hospitals used a one-way flow. Post-training 3 (25%) other hospitals had followed this recommendation and changed the flow of instruments. At two of these three hospitals dirty instruments were correctly passed through existing doors/windows into decontamination areas instead of being carried through clean areas. At the third hospital staff covered contaminated items during transport post training as participants had been taught. Pre-training instruments were often left to soak in chlorine in the operating room for long periods, until someone was available to clean them. Post-training personnel from 2 (17%) hospitals noted that instruments were moved to the decontamination area immediately after surgery and 3 (25%) hospital’s personnel noted they now removed gross soil from dirty instruments within one hour of use.

#### Cleaning

Pre-training, scheduled cleaning of SP areas was practiced in 5 (42%) hospitals, while post-training scheduled cleaning had improved to 11 (92%). 3 (25%) hospitals restricted access to the SP area pre-training, which improved to 6 (50%) hospitals post-training. At one facility it was reported that the cover on the counter used to dry clean instruments before sterilization was replaced after each procedure post-training. Issues with implementing training recommendations identified at one hospital was that heating water for cleaning took extra time and therefore this step in the cleaning process tended to be eliminated. At yet another hospital the water distiller had broken and workers were using saline solution, detrimental to stainless steel, to clean steel instruments.

#### Inspection and packaging

Inspection of instruments pre-training was practiced in 1 (8%) hospital, improving to 9 (75%) hospitals post-training. Hospital staff were noted to be spending more time inspecting instruments carefully, disassembling them for cleaning, and reassembling them for packaging. At 2 (17%) hospitals closer inspection was required as instruments were found to have bioburden on them post-cleaning. Pre-training appropriate instrument wrapping materials (either cloth or metal containers) were available at 9 (75%) of hospitals, whereas post training 11 (92%) were using appropriate protective packaging methods. Wrapping materials were found to be mostly of cloth, but occasionally paper was used. At one hospital wrapping materials were unavailable.

#### Sterilization and storage

Chemical indicator tape turns color once exposed to steam, and is used to identify packages that have been through the sterilization process. Pre-training 5 (42%) hospitals used chemical indicator tape, while post-training 8 (67%) were using the tape. Pre-training 8 (67%) hospitals had functioning autoclaves, and 10 (83%) hospitals had functioning autoclaves post-training. Tap water, not recommended for autoclaves, was still used for the majority of autoclaves as distillers were unavailable. One hospital had a table top sterilizer on the floor, while at another the dry heat sterilizer was placed on wood pallets, not recommended due to their capacity to retain bacteria and unstable support for the machine. Post training 3 (25%) hospitals did not have fully functioning autoclaves and at one hospital staff had resorted to boiling instruments as their autoclave, working pre-training, had broken and needed repair. One hospital indicated that they could only use their autoclave if there was good electricity as their generator was not powerful enough to run the autoclave. Sterilized instruments were moved to an enclosed area for storage at 7 (58%) hospitals pre-training, and 8 (67%) post-training.

#### Education

Posters were noted to be placed on walls providing instruction to staff at 2 (17%) hospitals pre-training and 9 (75%) hospitals post-training. However, where there had been educational posters on the wall of one hospital they had been removed before the follow-up assessments were completed. It is unclear as to why the posters were removed. Additional SP training had been implemented at all 12 hospitals by participants who received the ToT component of the program. The number of additional HCWs trained ranged from 3 to 72, totaling 254 additional staff trained in SP practices following SPECT’s course. Whether this amount of training is sustainable is not known given the time limitations of this research project.

## Discussion

Our study shows that a training program in SP increased participants knowledge of SP and resulted in improvements to SP practices in their work settings at four and six months, respectively. These findings provide evidence that with minimal investment in resources, practice changes can occur with guidance and support from knowledgable workers. Incorporating a ToT course into education sessions is one way of ensuring sustainability of this innovative approach to decreasing surgical risks.

Hospital assessments identified changes to SP practices, as well as barriers to changes that involved both lack of resources and organizational resistance to change. Indicators where no change occurred involved resources, or lack thereof. Increasing access to proper cleaning solutions, as well as indicators for verifying sterilization of instruments, is required to improve SP practices. One of the issues identified was a lack of awareness of basic tools required for improved cleaning and decontamination. With the training, participants became aware of the difference brushes and cleaning solutions make in instrument cleanliness and usability. The SPECT educator’s follow up meetings with administrators and attempts to access basic cleaning supplies for hospitals resulted in increased demand for these products by administrators. However, as the Ethiopian ministry of health had a central supply depot the government needed to consider changing their supply acquisition and communicating with hospitals that these supplies were now readily available. This did not occur during the course of this research project.

Of note are two indicators that had poorer results post assessment than identified during the pre-assessment. The change in enclosed storage of surgical instruments post sterilization and not moving cleaned instruments out of the decontamination area for packaging occurred at one hospital where there was significant resistance to change. Not only did they not extend education and training, but they firmly resisted removing chlorine solution from their cleaning processes. With resistance to change it appears there was also a negative attention to the details of safe instrument processing, where instruments were not moved to a clean area for packaging post cleaning and were stored in an open cupboard under open windows, increasing the risk of contamination. Future training needs to consider how to address issues of resistance with supportive encouragement for positive small changes. Of note, these indicators may also have been impacted by individual employee practice, with a lack of standard operating procedures that are followed by all employees. Consideration to implementing standard operating procedures in sterile processing would be beneficial for all health care facilities as a means of ensuring safe practice.

While some infrastructure changes were made by several of the facilities, the majority needed to work with existing structures and had little flexibility to make required structural changes. Ensuring new facilities or renovations to existing facilities consider SP practices and flow when working to address surgical capacity and hospital improvements is critical in establishing staff safety and improved patient outcomes.

One of the biggest challenges was the removal of 0.5% chlorine solution from the SP decontamination practice. The Ethiopian Infection Prevention and Patient Safety (IPPS) guideline required instruments be soaked in 0.5% chlorine solution immediately after use. The WHO 2016 global guidelines -[3]- states that surgical instruments are not recommended to be soaked in any disinfectant, including 0.5% chlorine, as it is corrosive to steel instruments, may be inactivated by blood and body fluids resulting in microbial contamination, poses a risk to health care workers, and may lead to antimicrobial resistance to the disinfectant (p.52). SPECT incorporated these guidelines into the SP training in Ethiopia. Staff at three (25%) hospitals were concerned that their personal safety would be impacted by the removal of 0.5% chlorine solution–believing use of chlorine decreased their risk of contracting HIV. When hospitals removed 0.5% chlorine solution the Regional Infection Prevention auditing team gave them a poorer ranking as they were not following the national guidelines. Paying attention to not only the processes which represent improvement in practice, but also the larger regulating documents needs to be part of implementing change. In follow up, SPECT’s educator collaborated with the Ethiopian government to update parts of the national IPPS guidelines as well as have the government representatives write a letter of permission to the SS2020 facilities with permission to remove the 0.5% chlorine solution from their SP practice.

Our study has some limitations. We were not able to correlate the improvement in practice with a decrease in surgical site infections due to the lack of patient record keeping at the facility level. Also, the fact that this was a study involving only 12 hospitals creates a seemingly greater improvement when looking at percentage growth, possibly over-emphasizing the significance of the intervention. A further limitation is that since the post-assessment was completed less than a year after training the long-term durability of the changes is unknown.

Of note also is the inability to control for confounding variables before or after the study, such as access to resources, competing interventions, or structural changes. Due to small sample size generalisability of the findings is not possible, and therefore we recommend more studies in various LMICs be conducted to increase confidence in study results. It may be beneficial in the future to conduct a randomised trial–providing education for all facilities and randomly including mentoring and hospital visits to half the hospitals–in order to gain a greater understanding of the benefits of on-site hospital and participant support in ensuring long-term sustainability of the project.

## Conclusion

Increased attention to and knowledge in SP practices and care of instruments with a short, one-week education course provides evidence that applying resources to a largely under-resourced area of healthcare can result in decreased risks to patients and staff. Providing education in SP and ensuring staff have the ability to disseminate their learnings to other HCWs results in decreasing risks to patients. Ensuring that standard operating procedures for SP are available, that all HCWs are knowledgeable in the procedures to be followed and understand the rationale behind them, will increase adherence to safe practice. Updating national guidelines when necessary will ensure best practice is being endorsed and will also support HCWs to keep themselves and patients safe.

We have shown what can be done to make changes in SP practices in one LMIC. We have shown that participants improved their SP practices, which they shared with others working in their hospitals. Based on these findings, we recommend that others involved in the Global Surgery 2030 initiative [17] include assessments and interventions in SP practices as an integral part of that initiative.

## Supporting information

S1 Appendix Hospital assessment form. (PDF)

S2 Appendix Pre-post test. (PDF)

S3 Appendix Hospital pre-assessment summary data set. (XLSX)

S4 Appendix Pre-post test results summary data set. (XLSX)

S1 File ARIC adapted pre-post questionnaire. (PDF)

S2 File BMJ adapted hospital assessment form. (PDF)
