# Supplementary material for: The impact of a short-term training program on workers’ sterile processing knowledge and practices in 12 Ethiopian hospitals: A mixed methods study
Source: PLoS One. 2019 May 1;14(5):e0215643. doi: 10.1371/journal.pone.0215643 (PMC6493726; doi:10.1371/journal.pone.0215643)
Supplement: S1 Appendix — (PDF) [file pone.0215643.s001.pdf]

Medical Device Reprocessing (MDR) Hospital Assessment Form

Date (mm/dd/yyy):___________________________________
Name of Hospital:____
Name of Hospital Director: ____
Contact information for Hospital Director: ____

Name of person responsible for MDR: ____
Job Title: ____
Contact Information: ____

General Hospital and Staff Information	Number		Comment
Number of operating rooms			
Average number of surgeries performed weekly.			
Number of staff involved in MDR			
Number of staff who have received classroom training in MDR			
Number of staff who have received on-the-job training in MDR			

MDR Setting	Yes	No	Comment
Are the walls painted with a washable paint?			
Is the MDR area cleaned on a scheduled rotating basis?			
Are sharps placed in a sharps container after use?			
Are sharps incinerated on the premises?			
Are there signs restricting access of unauthorized staff to the MDR area?			

Infection Control - Staff	Yes	No	Comment
Do MDR Staff wear PPE?			
If yes, what type of PPE is used by MDR staff?			
gloves			
gowns			
head covers			
protective eye wear			
masks			
booties			

Are hand washing stations available for MDR staff?			
Is soap available for handwashing for MDR staff?			
Is running water available for handwashing for MDR staff?			
Are gloves changed as needed?			
Do contaminated instruments flow from dirty to clean areas in the MDR process?			
Are medical devices passed through a window to the decontamination area post surgery?			

If no window is available, are contaminated items covered during transport to the decontamination area?			
Is the decontamination room separate from the packaging area?			
Is a sharps container in use in the decontamination area?			
Are there posters on the wall providing instruction to MDR staff?			

**Cleaning and Decontamination Process**	**Yes**	**No**	**Comment**
Are instruments soaked in soap and water immediately after use in the OR?			
If yes to the above question, are instruments soaked for less than 1 hour after use?			
If no to the above question, are instruments soaked in a chlorine solution?			
Are enzymatic detergents used to clean instruments?			
Are instruments brushed under water in a second bowl of soap and water after initial decontamination?			
Are instruments rinsed with clean/distilled water after removal of soil?			
Are instruments thoroughly dried with a clean, soft cloth after rinsing?			
Is more then one sink used in the decontamination and cleaning process?			
Are clean brushes in good condition in use in the decontamination area?			
Do MDR staff have access to clean, lint free cloths, various sized brushes, syringes, and sponges for cleaning?			
Are biocidal detergents available to clean tools used in decontamination and cleaning each shift?			

**Packaging and Inspection**	Yes	No	Comment
Are instruments moved to a clean area with no dirty instruments after cleaning and drying?			
Are instruments carefully inspected prior to packaging			
Are instruments dissasembled for cleaning and reassembled in the packaging area?			
Are instruments packaged with a protective cover? (i.e. wrapped in permeable cloth and placed in metal box)?			
Are instrument sets labeled following packaging?			

**Sterilization**	Yes	No
Is a functioning autoclave being used to sterilize instruments?		

Is a dry heat sterilizer used to sterilize instruments?			
Is the autoclave/dry heat sterilizer in a room separate from the decontamination area?			
Are containers used in the autoclave covered with porous cloth or do they contain vents?			
*Comment on length of time and temperatures used to sterilize instruments			
Are chemical indicators being used? How frequently?			
Are biological indicators being used? How frequently?			
Are times posted indicating when instruments were placed in sterilizers?			

**Storage of medical devices**	Yes	No	Comment
Are instruments stored in metal boxes?			
Are instrument containers wrapped with cloth?			
Are instruments stored in an enclosed area after sterilization?			
Are sterilized instruments stored away from decontaminated areas?			

**Transport to OR**	Yes	No	Comment
Are sterilized instruments transferred to the OR in a covered trolley?			
Are sterilized instrumens carried to the OR by staff?			
