# Supplementary material for: The impact of a short-term training program on workers’ sterile processing knowledge and practices in 12 Ethiopian hospitals: A mixed methods study
Source: PLoS One. 2019 May 1;14(5):e0215643. doi: 10.1371/journal.pone.0215643 (PMC6493726; doi:10.1371/journal.pone.0215643)
Supplement: S1 File — (PDF) [file pone.0215643.s005.pdf]

Appendix A

Infection Prevention Test

Personal Information

Name ____

Age: ___ 18-30 ___ 31-50 ___ 51 -65 ___ over 65

Contact Information

Address ________________________________

Phone Number ________________________

e-mail Address ________________________

Education: I have completed:

Elementary School

High School

University

Number of years you have worked in a medical device reprocessing area: ________________

Circle the one best answer based on your understanding.

1. The main way microorganisms are spread through hospitals is:
   a. Hands.
   b. Poor ventilation.
   c. Brushes used for cleaning.
   d. Surgical instruments that are not sterilized.

2. Gloves are useful to:
   a. Protect patients.
   b. Protect employees.
   c. Prevent cross contamination of instruments.
   d. All of the above.

3. When using hand sanitizer or washing your hands, which area of the hand is most often missed?
   a. Palm.
   b. Thumbs.
   c. Back of hand.
   d. Interdigital spaces.

4. Characteristics of microorganisms include:

a. They can be easily destroyed.
b. They can be seen with the naked eye.
c. They are always dangerous to people.
d. They can be both beneficial and harmful to humans.

5. How are microorganisms destroyed?

a. By sterilizing them.
b. By disinfecting them.
c. By keeping them in a warm place.
d. By washing them with soap and water.

6. A person can acquire an infectious disease through:

a. Touching someone who has a disease.
b. Touching contaminated instruments with their bare hands.
c. Breathing the same air as someone who has an infectious disease.
d. All of the above.

7. A nosocomial infection refers to an infection that a person:

a. Is not able to fight.
b. Contracts in the hospital.
c. Spreads to others in the community.
d. Contracts before coming to the hospital.

8. Infection sources can include:

a. Water.
b. People.
c. Animals.
d. All of the above.

9. Cleaning and disinfecting are both important steps in reprocessing of medical devices. What is the main difference between the two processes?

10. Over dilution of a disinfectant can increase the microorganism's resistance.

Circle one: True or False

11. Circle two types of disinfectants that are considered 'high level' disinfectants.

Formaldehyde Glutaraldehyde Alcohol Sodium Hypochlorite
Hydrogen Peroxide Peracetic Acid

12. A disinfectant can be applied to objects in different ways, including:

  a. Wiping.
  b. Rubbing.
  c. Immersion.
  d. All of the above.

13. Effective steam sterilization is achieved only when instruments:

  a. Are disassembled.
  b. Are in open position.
  c. Are completely clean.
  d. All of the above.

14. Choose the preferred sterilization method for surgical instruments.

  a. Dry heat.
  b. Autoclave.
  c. Steam Sterilizer.
  d. All of the above.

15. Sterilized items can become unsterile if they are:

  a. Moist.
  b. Dusty.
  c. Damaged.
  d. All of the above.
