# Supplementary material for: The impact of a short-term training program on workers’ sterile processing knowledge and practices in 12 Ethiopian hospitals: A mixed methods study
Source: PLoS One. 2019 May 1;14(5):e0215643. doi: 10.1371/journal.pone.0215643 (PMC6493726; doi:10.1371/journal.pone.0215643)
Supplement: S2 File — (PDF) [file pone.0215643.s006.pdf]

Sterile Processing Assessment and Check List

Date (mm/dd/yyy): ___________________________________
Name of Hospital: ____
Name of Hospital Director: ____
Contact information for Hospital Director: ____

Head of Sterile Processing (SP) services: ____
Job Title: ____
Contact Information: ____

**General Hospital and Staff Information**	**Number**
Number of operating rooms	
Average number of surgeries performed weekly	
Number of staff involved in SP	
Number of staff who have received classroom training in SP	
Number of staff who have received on-the-job training in SP	

List any organizations that have provided previous SP training: ____

**SP Infection Control & General Info**	**Yes**	**No**
SP Staff wear PPE when cleaning contaminated items		
The following type of PPE are available to SP staff:		
gloves		
gowns		
head covers		
protective eye wear		
masks		
booties		
Soap is available to SP staff for handwashing		
Clean running water available for handwashing		
The SP areas are cleaned on a scheduled rotating basis		
There are sharps containers available		
Sharps are incinerated on the premises		
There are signs restricting access of unauthorized staff to the SP area		
There are educational posters on the wall which provide instruction to SP staff		

**Transport of contaminated items from Point of Use areas**	**Yes**	**No**
Contaminated instruments flow from dirty to clean areas during transportation without cross contamination		
Contaminated items are covered during transportion from the theatre to the decontamination area		
Disposable sharps are removed by the Theatre Nurse at point of use		

Instruments are transported from theatre to the cleaning area immediately after surgery		

**Cleaning and Decontamination Process**	**Yes**	**No**
The decontamination area is physically separate from the packaging/sterilization area		
Visible gross soil, such as blood, is removed from the instruments within 1 hr after surgery		
Instruments are **not** soaked in a chlorine solution for more then 10 minutes		
Enzymatic cleaning detergents are available and used to clean instruments		
Instruments are scrubbed in a second sink/bowl of clean water and soap after removing visible bioburden in the first sink		
Instruments are rinsed with clean/distilled water after all visible and nonvisible soil is removed		
Instruments are thoroughly dried with a clean cloth after they've been cleaned and rinsed		
More then one sink used in the cleaning process		
The cleaning brushes being used are in good condition		
MDR staff have access to cleaning tools such as cloths, various sized brushes, syringes, and sponges		
Biocidal disinfectants available and used as part of the cleaning process		

**Packaging and Inspection**	**Yes**	**No**
Instruments are moved to a clean area, away from any risk of contamination, after the cleaning process is complete		
Instruments are carefully inspected prior to packaging		
Instruments are dissasembled for cleaning and reassembled for sterilization in the packaging area		
Instruments are packaged with a protective cover, such as cloth, or placed inside a rigid container		
After the instruments are packaged, the package is labeled		

**Sterilization**	**Yes**	**No**
A functioning steam sterilizer is being used to sterilize instruments		
A functioning dry heat sterilizer is being used to sterilize instruments		
The autoclave/dry heat sterilizer is in a room separate from the cleaning/decontamination area		
Appropriate packaging material is used to protect the items being sterilized by steam/dry heat		
*Comment on length of time and temperatures used to sterilize instruments		
Chemical indicators are being used at least weekly		

Biological indicators being used at least monthly		
A record sheet is available to track the instruments going in and out of the sterilizer		

**Sterile Storage of medical devices**	**Yes**	**No**
Instruments are stored in an enclosed cabinet or room after sterilization		
Sterilized instruments are stored away from decontamination area and away from open windows		

Comments
